# Construction and Application of Elastin Like Polypeptide Containing IL-4 Receptor Targeting Peptide

**DOI:** 10.1371/journal.pone.0081891

**Published:** 2013-12-10

**Authors:** Vijaya Sarangthem, Eun A. Cho, Sang Mun Bae, Thoudam Debraj Singh, Sun-Ji Kim, Soyoun Kim, Won Bae Jeon, Byung-Heon Lee, Rang-Woon Park

**Affiliations:** 1 Department of Biochemistry and Cell Biology, Cell & Matrix Research Institute, Kyungpook National University, School of Medicine, Daegu, Republic of Korea; 2 Department of Nuclear Medicine, Kyungpook National University, School of Medicine, Daegu, Republic of Korea; 3 Division of NanoBioTechnology, Laboratory of Biochemistry and Cellular Engineering, Daegu Gyeongbuk Institute of Science and Technology, Daegu, Republic of Korea; Bascom Palmer Eye Institute, University of Miami School of Medicine, United States of America

## Abstract

Various human solid tumors highly express IL-4 receptors which amplify the expression of some of anti-apoptotic proteins, preventing drug-induced cancer cell death. Thus, IL-4 receptor targeted drug delivery can possibly increase the therapeutic efficacy in cancer treatment. Macromolecular carriers with multivalent targeting moieties offered great advantages in cancer therapy as they not only increase the plasma half-life of the drug but also allow delivery of therapeutic drugs to the cancer cells with higher specificity, minimizing the deleterious effects of the drug on normal cells. In this study we designed a library of elastin like polypeptide (ELP) polymers containing tumor targeting AP1 peptide using recursive directional ligation method. AP1 was previously discovered as an atherosclerotic plaque and breast tumor tissue homing peptide using phage display screening method, and it can selectively bind to the interleukin 4 receptor (IL-4R). The fluorescently labeled [AP1-V_12_]_6_, an ELP polymer containing six AP1 enhanced tumor-specific targeting ability and uptake efficiency in H226 and MDA-MB-231 cancer cell lines *in vitro*. Surface plasmon resonance analysis showed that multivalent presentation of the targeting ligand in the ELP polymer increased the binding affinity towards IL-4 receptor compared to free peptide. The binding of [AP1-V_12_]_6_ to cancer cells was remarkably reduced when IL-4 receptors were blocked by antibody against IL-4 receptor further confirmed its binding. Importantly, the Cy5.5-labeled [AP1-V_12_]_6_ demonstrated excellent homing and longer retention in tumor tissues in MDA-MB-231 xenograft mouse model. Immunohistological studies of tumor tissues further validated the targeting efficiency of [AP1-V_12_]_6_ to tumor tissue. These results indicate that designed [AP1-V_12_]_6_ can serve as a novel carrier for selective delivery of therapeutic drugs to tumors.

## Introduction

Targeted macromolecular polymer carriers offer the potential of effective drug delivery by virtue of their ability to decrease the rate of drug clearance after systematic administration and improve the plasma half-life of drugs [1]. One such engineered biopolymer is elastin-like polypeptide (ELP), which is an emerging drug carrier under development for cancer therapy. In several cancer models, ELPs have already been used for targeted delivery of small molecules drugs (doxorubicin) [2], [3], therapeutic peptides (c-Myc inhibitory peptide) [4] and proteins [5]. They have unique characteristics compared to other polymeric drug delivery systems, including low toxicity, good biodegradability, and biocompatibility [6], [7]. ELP biopolymers are derived from the structural motif found in mammalian elastin protein, which consists of the pentapeptide repeat, Val-Pro-Gly-Xaa-Gly (VPGXG), where Xaa (“guest” residue) can be any amino acid except proline [8], [9]. ELP polymers can be synthesized at the genetic level using recombinant DNA methods; thus, their sequence, composition, and molecular weight can be controlled. Accordingly, the hydrophobicity and degree of ionization can be precisely tuned for proper tissue distribution and subcellular uptake [10], [11]. Moreover, the numbers of targeting peptides or specific reactive sites for drug conjugation can be incorporated at the genetic level along with the ELP sequence, which is one of the difficulties to accomplish using synthetic polymers. ELPs are soluble in aqueous solutions below their transition temperature (T_t_), but hydrophobically collapse and aggregate at temperatures greater than T_t_ [12], [13]. This inverse transition temperature is fully reversible. The transition temperature of ELP can be controlled by varying the guest residue, molecular weight and concentration [10]. Notably, ELPs can be easily expressed and purified at high yield simply by exploiting inverse temperature cycling (ITC) method [14], [15].

Using phage display screening, we previously discovered an atherosclerotic plaque and breast tumor tissue homing peptide, CRKRLDRN, termed AP1 peptide that selectively binds to the interleukin -4 receptors (IL-4Rs) [16]–[18]. IL-4Rs are highly expressed in a wide variety of human tumors, including renal cell carcinoma, squamous cell carcinoma of the head and neck, malignant glioma, AIDS-associated Kaposi's sarcoma and breast cancer cell lines [19]–[22]. It has been reported that IL-4/IL-4R interactions amplify the expression of some anti-apoptotic proteins, including PED/PEA15 (15 kDa phosphoprotein enriched in astrocytes), cFLIP/CFLAR (CASP8 and FADD-like apoptosis regulator), and the BCL family proteins Bcl-xL and Bcl, thereby preventing drug-induced cancer cell death [23], [24]. Thus, targeting the IL-4R could possibly increase the therapeutic efficacy of anticancer drugs. Even though AP1 peptide possesses high target-specificity and efficient tumor-homing, it has limitation due to easy elimination from the circulation and short half-life.

The aim of this study is to increase the stability and binding affinity of the AP1 peptide *in vivo*, and thus increase its utility, through multivalent presentation of the AP1 peptide in an ELP polymer backbone. Many studies have demonstrated that the presentation of multiple targeting moieties on a polymer backbone can improve binding avidity and specificity compared to monovalent ligand presentation [25]. Accordingly, we designed and prepared a series of an AP1 peptide containing ELP polymer using recursive directional ligation (RDL) method and analyzed the characteristics and the efficacy *in vitro* and *in vivo*. The binding specificity of [AP1-V_12_]_6_ polymer towards cancer cells was analyzed using confocal microscopy and flow cytometry, and *in vivo* optical imaging in a tumor xenograft mouse model. The studies have revealed that the incorporation of multivalent targeting peptide ligand into ELP polymer facilitated the greater targeting to IL-4R expressing cancer cells.

## Materials and Methods

### Media and cell culture

H226 (human lung cancer cells), MDA-MB-231 (human breast cancer cells), and H460 (human lung cancer cells) were obtained from American Type Culture Collection (ATCC). H226 and H460 cells were grown in RPMI-1640 (Hyclone, Thermo Scientific, Logan, UT), and MDA-MB-231 cells were grown in Dulbecco's modified Eagle's medium (DMEM) (Hyclone, Thermo Scientific) supplemented with 10% fetal bovine serum (Hyclone, Thermo Scientific) and 100 U/mL penicillin and 100 µg/mL of streptomycin (Sigma Aldrich, St. Louis, MO, USA). Cells were maintained at 37°C in a humidified atmosphere containing 5% CO_2_.

### Designing of monomer gene and oligomerization

Synthetic oligonucleotides encoding genes of [VGVPG]_14_ and VGRKRLDRNG[VGVPG]_12_ referred as V_14_ and AP1-V_12_ were designed to contain *Bam*H I and *Hin*D III compatible cohesive ends upon annealing, which allows the annealed product to be directly ligated into a *Bam*H I and *Hin*D III-cleaved pRSET B+ vector (Invitrogen, CA, USA) [26], [27]. The resulting vector containing V_14_ and AP1-V_12_ were used as a monomer to synthesized [V_14_]_2_ and [AP1-V_12_]_2_. Additional rounds of RDL were performed to acquire different length [AP1-V_12_]_n_ polymers where n = 2, 4, 5 and 6 with different molecular weights and thermal transition characteristics. The polymers used in the study have the composition [V_14_]_6_ and [AP1-V_12_]_6_.

### [V_14_]_6_ and [AP1 -V_12_]_6_ proteins expression

The pET 25 b+ (Novagen, Canada, USA) expression plasmid was doubled digested with *Nde* I and *Hin*D III and purified using a gel extraction kit (ELPIS Biotech, Daejeon, Korea). The synthetic oligonucleotides 5′-TAT GAG CGG GCC GGG CTG GCC GTG CTA AA-3′ (sense) and 5′-AGC TTT TAG CAC GGC CAG CCC GGC CCG CTC A-3′ (antisense) were annealed to form double-stranded DNA containing *Nde* I, *Sfi* I, and *Hin*D III restriction sites and ligated to *Nde* I and *Hin*D III double-digested pET 25 b+ vector. After confirmation by DNA sequencing, the modified pET 25b+ vector was linearized with *Sfl* I and enzymatically dephosphorylated with CIP (New England Biolab, Ipswich, MA). The [V_14_]_6_ and [AP1-V_12_]_6_ genes were ligated to linearized, modified pET 25b+ vector and transformed into DH5α competent *E. coli* cells. Plasmids with the respective gene ligations were confirmed by restriction digestion with *Nde* I and *Hin*D III (New England Biolab), followed by gene sequencing (Macrogen Inc., Seoul, Korea).

### ELP protein purification

BL21 (DE3) chemically competent *E. coli* cells (Invitrogen) were further transformed with modified pET 25b+ vector containing [V_14_]_6_ and [AP1-V_12_]_6_ gene for protein expression. Starter cultures were prepared by inoculating 10 mL of Circle grow media (MP Biomedicals, CA, USA) containing 100 µg/mL ampicillin (Sigma Aldrich, MO, USA) with the expression strain and incubating for 6 h at 37°C. Starter cultures were then inoculated into 1 L of fresh Circle grow media containing ampicillin and incubated for 12 h at 37°C. The cells were then harvested by centrifugation at 4000 rpm for 20 min at 4°C and suspended in 10 mL phosphate-buffered saline (PBS). Cells were lysed by sonication at 4°C, and ELP protein was purified using inverse transition cycling (ITC). Four rounds of ITC were performed to eliminate cell contaminants. ELP purity was checked by SDS-PAGE, followed by Coomassie blue staining (Bio-Rad, Hercules, CA). ELP concentration was measured by Cary UV-visible spectrophotometer (Agilent Technologies, CA, USA) using an extinction coefficient of 5690 M^−1^ cm^−1^ for both [V_14_]_6_ and [AP1-V_12_]_6_.

### Thermal characterization

Transition temperature (T_t_) of [V_14_]_6_ and [AP1-V_12_]_6_ were determined by monitoring the turbidity profile of protein solutions at wavelength 350 nm as a function of temperature using Cary UV-visible spectrophotometer equipped with temperature controller (Agilent Technologies). The absorbance was monitored from 20°C to 45°C in 1°C/min increments. The T_t_ of [V_14_]_6_ and [AP1-V_12_]_6_ protein were determined at a concentration of 10 µM.

### Fluorophore conjugation

[V_14_]_6_ and [AP1-V_12_]_6_ proteins were labeled with Alexa Fluor 488-C5 maleimide (Invitrogen ) or Alexa 680 maleimide (BioActs, Incheon, Korea) dyes, as per company's protocol.

### Flow cytometry analysis

IL-4R highly expressed cancer cell lines such as H226 and MDA-MB-231 cells were used to examine the binding specificity of [AP1-V_12_]_6_ polymer. H460 cell was used as IL-4R negative control [28]. H226, MDA-MB-231, and H460 (2×10^5^) cells were incubated with 2% BSA for 30 min at 37°C to block the nonspecific binding and further incubated with 10 µM Alexa 488-labeled AP1 peptide, [V_14_]_6_ and [AP1-V_12_]_6_ proteins for 1 h at 4°C. The cells were washed twice with PBS, suspended in 200 µL of PBS, and analyzed by flow cytometry (BD Bioscience, San Jose, CA, USA). For analysis, 10,000 events were collected for each sample and the total percentage of Alexa 488 labeled polymer bound to cells was calculated by comparing with untreated cells.

### Competition assay

3×10^5^ of H226 and MDA-MB-231 cells were pretreated with IL-4R antibody (R&D systems, Canada, USA) at various concentrations such as 1, 5 and 10 µg at 4°C for 1 h. The cells were then incubated with 10 µM Alexa 488 labeled [AP1-V_12_]_6_ for 1 h at 4°C. After washing with PBS for two times, cells were suspended with 300 µl of PBS and analyzed by flow cytometry. 10,000 events were analyzed for each sample.

### Confocal Microscopy

H226, MDA-MB-231 and H460 cells were seeded on four chambered slide and grown to 80% confluence. Cells were then incubated with 10 µM Alexa 488 labeled [V_14_]_6_ and [AP1-V_12_]_6_ proteins and AP1 peptides for 1 h at 4°C and 37°C. Unbound peptides were washed out with PBS, and cells were fixed with 4% paraformaldehyde (Sigma Aldrich). Cell nuclei were stained with 4′,6-diamidino-2-phenylindole (DAPI; Sigma Aldrich), and chamber slides were mounted with anti-fade reagent (Invitrogen). Images were captured and analyzed in sequential scanning mode using a Zeiss LSM-510 Meta confocal microscope.

### Cell viability and proliferation assay

5×10^3^ of MDA-MB-231 cells were plated in serum containing media and incubated for 16 h. The cells were then treated with different concentration of [AP1-V_12_]_6_ and [V_14_]_6_ (1, 5, 10, 20 µM) in media for various time intervals such as 12, 24, 48, and 72 h respectively. WST-8 solution was added to each wells and further incubated for 1 h. WST-8 values (average absorbance, 450 nm) were measured at different time intervals to determine cell viability.

In order to access the effect of [AP1-V_12_]_6_ on IL-4 induced cell proliferation, MDA-MB-231 (2×10^3^) cells were serum starved for 16 h by plating in low serum (1% FBS) with or without different concentrations of IL-4. The cells were further cultured with low serum (1% FBS) media containing different concentration of IL-4 (10, 50, 100 ng/ml) and [AP1-V_12_]_6_ and [V_14_]_6_ (10 µM) for 24, 48, and 72 h. Cell proliferation was analyzed by measuring the WST-8 absorbance at 450 nm.

### Surface Plasmon Resonance (SPR) analysis

Interactions of AP1, [V_14_]_6_ and [AP1-V_12_]_6_ with the IL-4R were analyzed at 25°C using a surface plasmon resonance instrument (Reichert Life Sciences, NY, USA). IL-4Rα (CD124) (Sino Biological, Beijing, China) was immobilized by activating the carboxy methyl group on dextran-coated chips (Reichert Life Sciences) through a reaction with N-hydroxysuccinimide (Sigma Aldrich), followed by covalent bonding of the ligands to the chip surface via amide linkages and blocking of excess activated carboxyls with ethanolamine. Different concentrations of AP1 peptide (3.125 to 50 µM) and [AP1-V_12_]_6_ (125 nM to 1 µM) in binding buffer were allowed to flow over surfaces containing immobilized IL-4Rα (2000±400 RU) for 3 min at a rate of 25 µL/min. The sensor surface was regenerated after each association and dissociation cycle by injecting 10 mM HCl for 10 s. The interaction of ligand and analyte was analyzed using Scrabber 2 Biologic software. Binding kinetics was assessed by determining association (k**_on_**), dissociation (k_off_), and equilibrium (K_D_) constants.

### Animal Model

This study strictly followed the recommendations of National Institute of Health (NIH) for the Care and Use of Laboratory Animals. Animal experiments were reviewed and approved by the Committee on the Ethics of Animal Experiments of the Kyungpook National University (Permit Number: KNU 2012-124). All efforts were made for minimizing animal suffering. Female nude mice (BALB/c nude; body weight, 20±3 g; n = 5) were housed in a specific pathogen-free environment at 22±2°C, 55±5% relative humidity with light. Tumors were generated by subcutaneously injecting MDA-MB-231 cells (5×10^6^ cells) into the right flank of 5-wk-old BALB/c nude female mice and allowing them to grow for 10days [17], [29]. For *in vivo* analysis of tumor targeting, [V_14_]_6_ and [AP1-V_12_]_6_ were labeled with Alexa 680 (Cy5.5) at the C-terminal Cys residue of the protein. Tumor-bearing mice were anesthetized under inhalational anesthesia (1%, w/v, isofurane in 2 L oxygen), then injected with Cy5.5-labeled [AP1-V_12_]_6_ (n = 5) or, [V_14_]_6_ control (n = 5) of approximately 8 mg/kg via the tail vein. *In vivo* fluorescence images were taken at different time intervals after anesthetization (10 min, 1 h, 6 h, 12 h, and 24 h) using an eXplore Optix system (ART Advanced research technologies Inc., Montreal, Canada).

### Tissue preparation

At 6 h time point, animals were euthanized with CO_2_, tumors and organs were removed from a subset of animals, and *ex vivo* fluorescence images were collected. The tumor tissues were then fixed by incubating with 4% paraformaldehyde overnight and frozen for cryosectioning. Tissue slices (8-µm thick) were incubated overnight at 4°C with anti-IL-4R antibody (R&D Systems; 1∶100) and then incubated for 30 min at room temperature with Alexa 488-labeled goat anti-mouse IgG secondary antibody (1∶200). After staining nuclei with DAPI, sections were slide-mounted and observed under a confocal microscope.

### Statistical analysis

The statistical significance was determined using Student's t-test for two groups and one-way ANOVA for comparing multiples groups. ***P<0.0001, **P<0.001, and *P<0.05 were considered as statistically significant and denoted by asterisks in the Figures.

## Results

### Design of [AP1-V_12_]_n_ genes and protein expression

**Figure 1:**
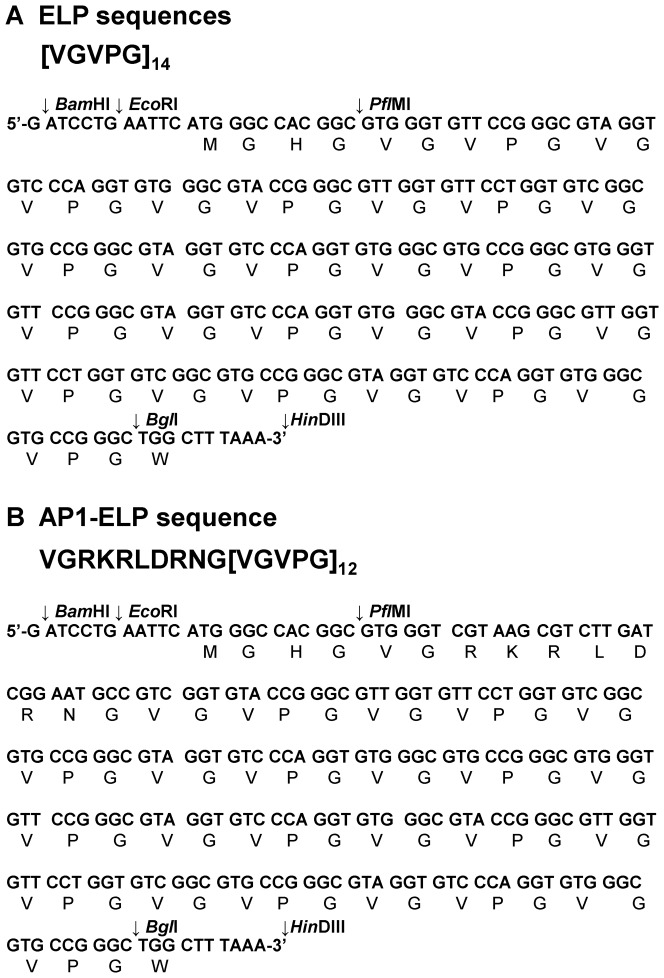
Monomer genes of V_14_ and AP1-V_12_. (A) [VGVPG]_14_ and (B) VGRKRLDRNG[VGVPG]_12_ monomeric genes and their corresponding polypeptide sequences.

We successfully constructed [V_12_]_n_ and [AP1-V_12_]_n_ gene libraries using the RDL method [11]. The ELP libraries consisted of the monomer genes repeat Val-Pro-Gly-Val-Gly with Valine at the guest residue of ELP pentapeptide (Fig. 1A), whereas the [AP1-V_12_]_n_ library contained modified AP1 sequence, in the N-terminal region of the ELP coding sequences (Fig. 1B). The monomer gene of [AP1-V_12_]_n_ was designed such that at each round of gene oligomerization by RDL, the AP1 sequence was repeated along with the ELP sequence. This iterative process yielded [V_12_]_n_ (Fig. S1A) and [AP1-V_12_]_n_ genes (Fig. S1B) with variable lengths and numbers of repeats. For subsequent *in vitro* and *in vivo* studies, we chose [AP1-V_12_]_6_ containing six AP1 sequence repeats as the target-specific polymer and [V_14_]_6_ as the non-targeting polymer control. For successful expression, [AP1-V_12_]_6_ and [V_14_]_6_ were ligated into a modified pET25b+ vector containing the trailer sequence WPC, providing a cysteine residue for conjugation of fluorophore or drug (Fig. S1C-E). The SGPG[VGRKRLDRNG(VGVPG)_12_]_6_WPC and

**Figure 2:**
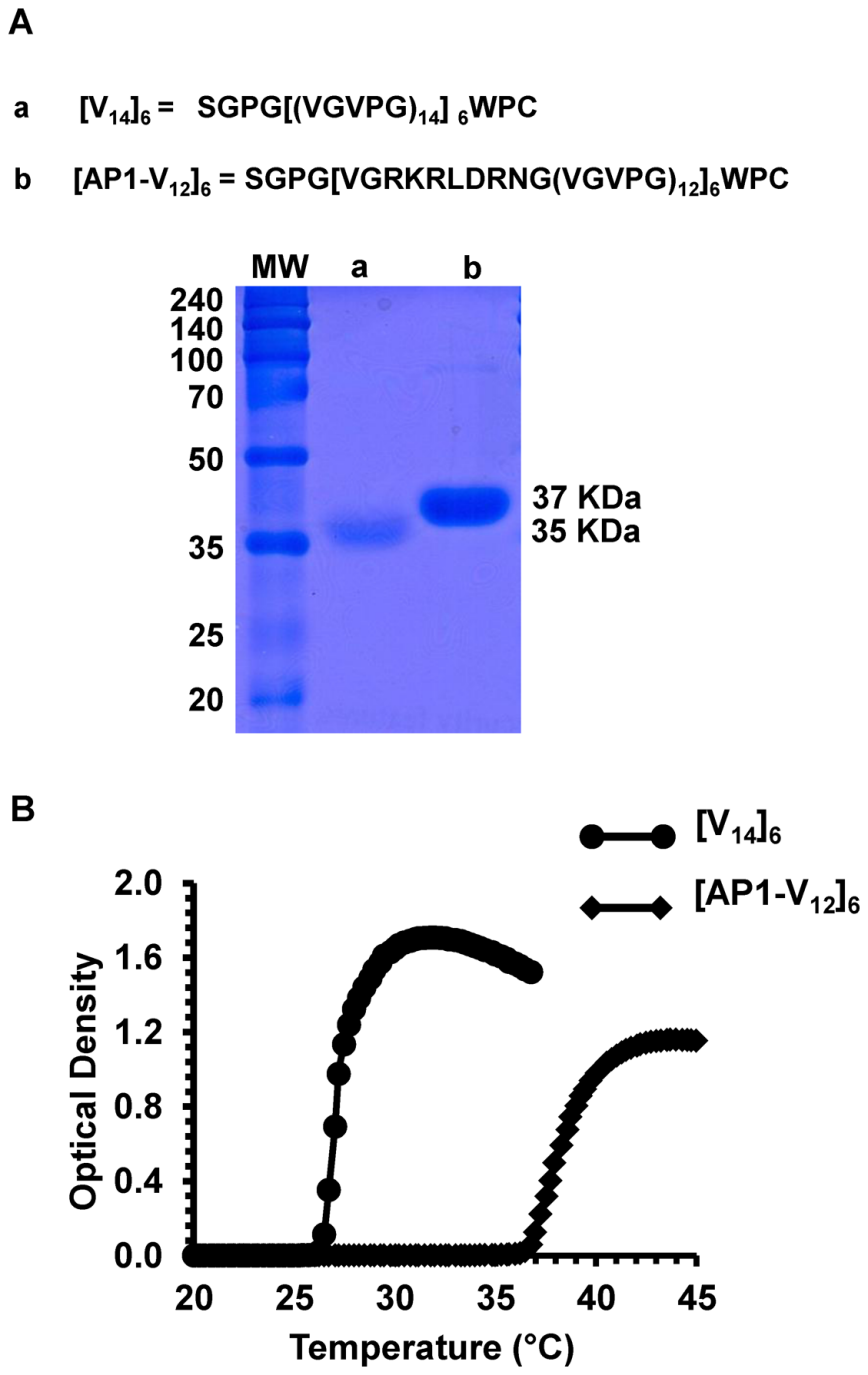
Protein expression and thermal characterization. (A) Polypeptide sequences of (a) [V_14_]_6_ and (b) [AP1-V_12_]_6_, expressed proteins were visualized by Coomassie Blue staining after SDS-PAGE analysis. Left lane: protein molecular weight marker (in kDa). Expected sizes of [V_14_]_6_ (∼35 kDa) and [AP1-V_12_]_6_ (∼37 kDa) are indicated on the right. (B) The turbidity profiles of [V_14_]_6_ and [AP1-V_12_]_6_ protein verses OD_350_ as a function of temperature (increased at a rate of 1°C min^−1^) were obtained.

 SGPG[(VGVPG)_14_]_6_WPC proteins were purified using the ITC method; the total yield was ∼20 mg/L. After four rounds of ITC, SDS-PAGE followed by Coomassie blue staining showed that [AP1-V_12_]_6_ and [V_14_]_6_ were approximately ∼37 kDa and ∼35 kDa in size, respectively, with minimal contamination (Fig. 2A).

### Thermal characterization

The T_t_ of [AP1-V_12_]_6_ and [V_14_]_6_ proteins were characterized at a concentration of 10 µM in PBS. The turbidity profiles were determined by measuring optical density at 350 nm (OD_350_) as function of temperature in 1°C min^−1^ increments. The T_t_, defined as the temperature at which turbidity in the protein solution reached 50%, was in the range of 37°C to 39°C for [AP1-V_12_]_6_ and 26°C to 28°C for and [V_14_]_6_ (Fig. 2B). Incorporation of the hydrophilic AP1 sequence into the ELP gene increased the T_t_ compared to the ELP control [30]. We observed that the inverse transition of the polymer [AP1-V_12_]_6_ is just above the physiological body temperature, which is preferable for clinical applications.

### *In vitro* cell binding of [AP1-V_12_]_6_ polymer

**Figure 3:**
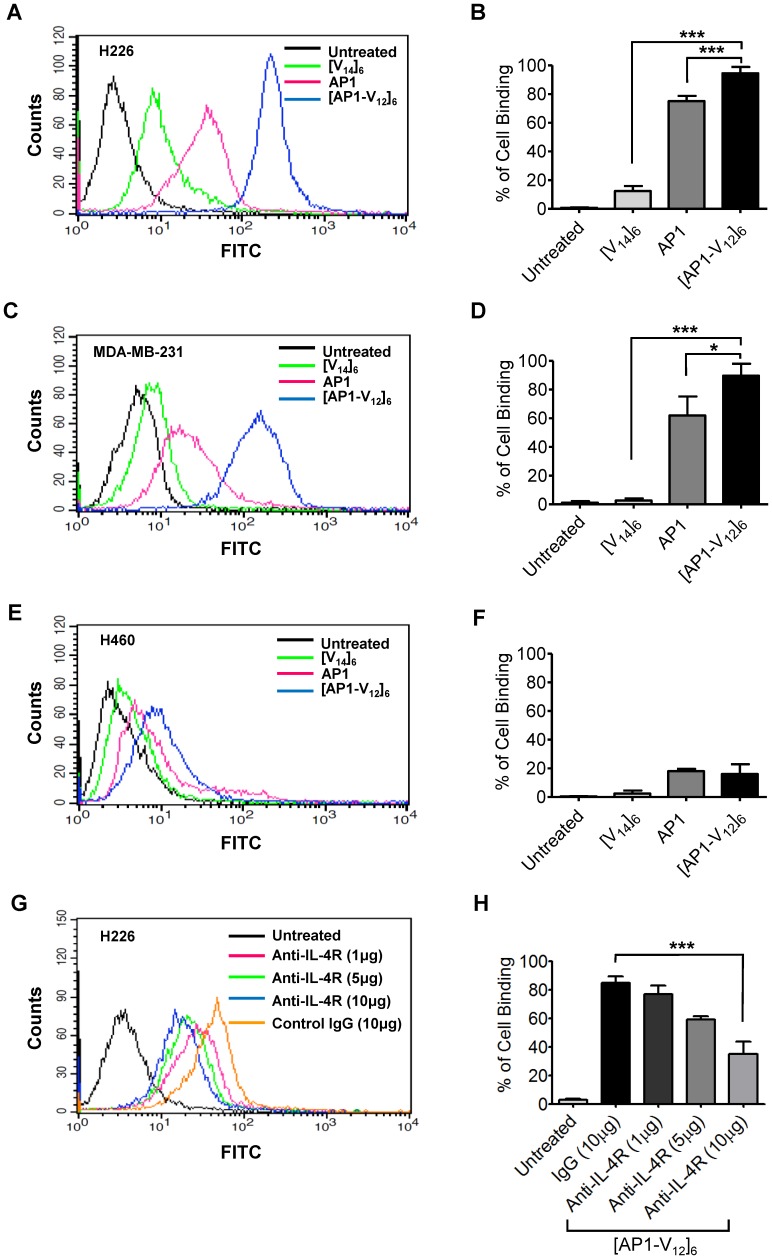
*In vitro* binding assays of [V_14_]_6_ and [AP1-V_12_]_6_ polymer. (A, B) H226, (C, D) MDA-MB-231 and (E, F) H460 cells were incubated with 10 µM of [V_14_]_6_, [AP1-V_12_]_6_ and AP1 for 1 h at 4°C. Cell binding was determined using flow cytometry. Histograms are representative of three independent experiments. Graphical bars (on right) represent the percent of Alexa 488 labeled polymer bound to cells as mean ±SD of data obtained from three separate experiments performed in triplicates. ***P<0.0001, **P<0.001, and *P<0.05, one-way ANOVA; n = 3. (G, H) H226 cells (3×10^5^ cells) were pre- incubated with different concentrations (1, 5 and 10 µg/mL) of anti-IL-4 receptor antibody followed by 1 h incubation with 10 µM Alexa-labeled [AP1-V_12_]_6_ at 4°C. The cells were further suspended in 300 µL of PBS after washing and analyzed using flow cytometry. Histograms are representative of three independent experiments. Graphical bars represent the percent of Alexa 488 labeled polymer bound to cells as mean ±SD of data obtained from three separate experiments performed in triplicates. ***P<0.0001, One way ANOVA; n = 3.

To study binding efficiency, we labeled the C-terminal Cys residue of [AP1-V_12_]_6_ and [V_14_]_6_ proteins with Alexa 488. FACS analysis confirmed that the cell-binding capacity of the targeted [AP1-V_12_]_6_ polymer in H226 (Fig. 3A, B) and MDA-MB-231 cells (Fig. 3C, D) after a 1-h incubation at 4°C was higher than that of the non-targeted [V_14_]_6_ polymer (P<0.0001). Both non-targeted [V_14_]_6_ and targeted [AP1-V_12_]_6_ polymers showed significantly lower binding towards H460 cells (Fig. 3E, F). For the [AP1-V_12_]_6_ polymer, cell attachment was 5.9±1.6 fold higher in H226 cells and 5.6±1.3 fold higher in MDA-MB-231 cells compared to H460 cells. In addition, the AP1 peptide showed 4.2±1.8 and 3.4±1.1 folds higher binding to H226 and MDA-MB-231 cells than to H460 cells. Thus, the [AP1-V_12_]_6_ polymer exhibited 1.7 and 2.2 fold higher cell-binding capacity in H226 (P<0.0001) and MDA-MB-231(P<0.05) cells than the targeting AP1 peptide alone. Our results suggested that multivalent presentation of the AP1 sequence in the [AP1-V_12_]_6_ polymer allowed greater accumulation on cells compared to the AP1 peptide.

The IL-4R dependence of [AP1-V_12_]_6_ polymer binding to tumor cells was further confirmed using competition assays. The binding of tumor cells by [AP1-V_12_]_6_ polymer was remarkably reduced in a concentration-dependent manner by pre-incubation with different concentrations (1, 5 and 10 µg/mL) of anti-IL-4R antibody (Fig. 3 G, H).

### Cellular localization of [AP1-V_12_]_6_ polymer and affinity for IL-4R

**Figure 4:**
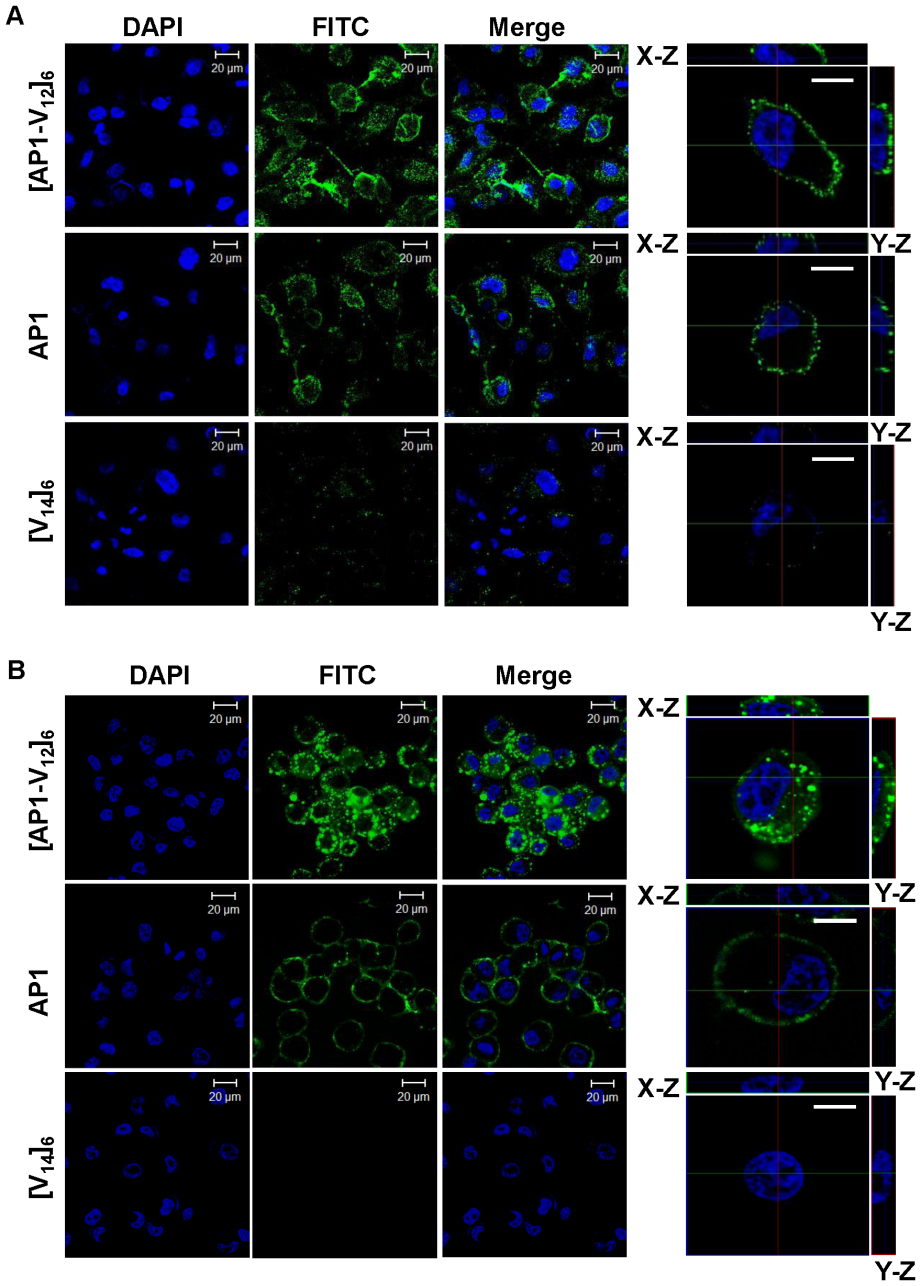
Analysis of cellular localization of [AP1-V_12_]_6_ polymer. Confocal laser scanning microscopic images of H226 cancer cells treated with 10 µM of [AP1-V_12_]_6_, AP1, or [V_14_]_6_ at (A) 4°C and (B) 37°C. Representative confocal images of three experiments (scale bar 20 µm). *Right panels*: Examination of [AP1-V_12_]_6_, AP1 and [V_14_]_6_ cellular location by Z-section scanning of confocal microscopic images. Representative confocal images of three experiments (scale bar 10 µm).

The cellular localization of [AP1-V_12_]_6_ polymer at different temperatures were further confirmed by confocal microscopic imaging, which clearly revealed that [AP1-V_12_]_6_ polymer and AP1 peptide accumulated more efficiently to the cell surface of H226 (Fig. 4A) and MDA-MB-231 (Fig. S2A) cells at 4°C compared to H460 cells (Fig. S3A). [AP1-V_12_]_6_ polymer was well internalized when incubated at 37°C whereas no uptake was seen in case of [V_14_]_6_ polymer in both H226 (Fig. 4B) and MDA-MB-231 cells (Fig. S2B). Both non-targeted [V_14_]_6_ and targeted [AP1-V_12_]_6_ polymers have shown lower cell binding and uptake in case of IL-4R negative H460 cells (Fig. S3A, B). Thus, these results indicated that cellular uptake of [AP1-V_12_]_6_ polymers were not due to nonspecific effects of increased temperature. Further evidence for cellular localization of [AP1-V_12_]_6_ polymer on the cells were obtained by exploiting confocal microscopic Z-series images captured in sequential scanning mode, showed that the [AP1-V_12_]_6_ polymer was well distributed on the plasma membrane of H226 cells at 4°C (Fig. 4A, right panel) and internalized into cytoplasm when temperature was increased to 37°C (Fig. 4B, right panel).

### Effect of [AP1-V_12_]_6_ polymer on proliferation of MDA-MB-231 cells

**Figure 5:**
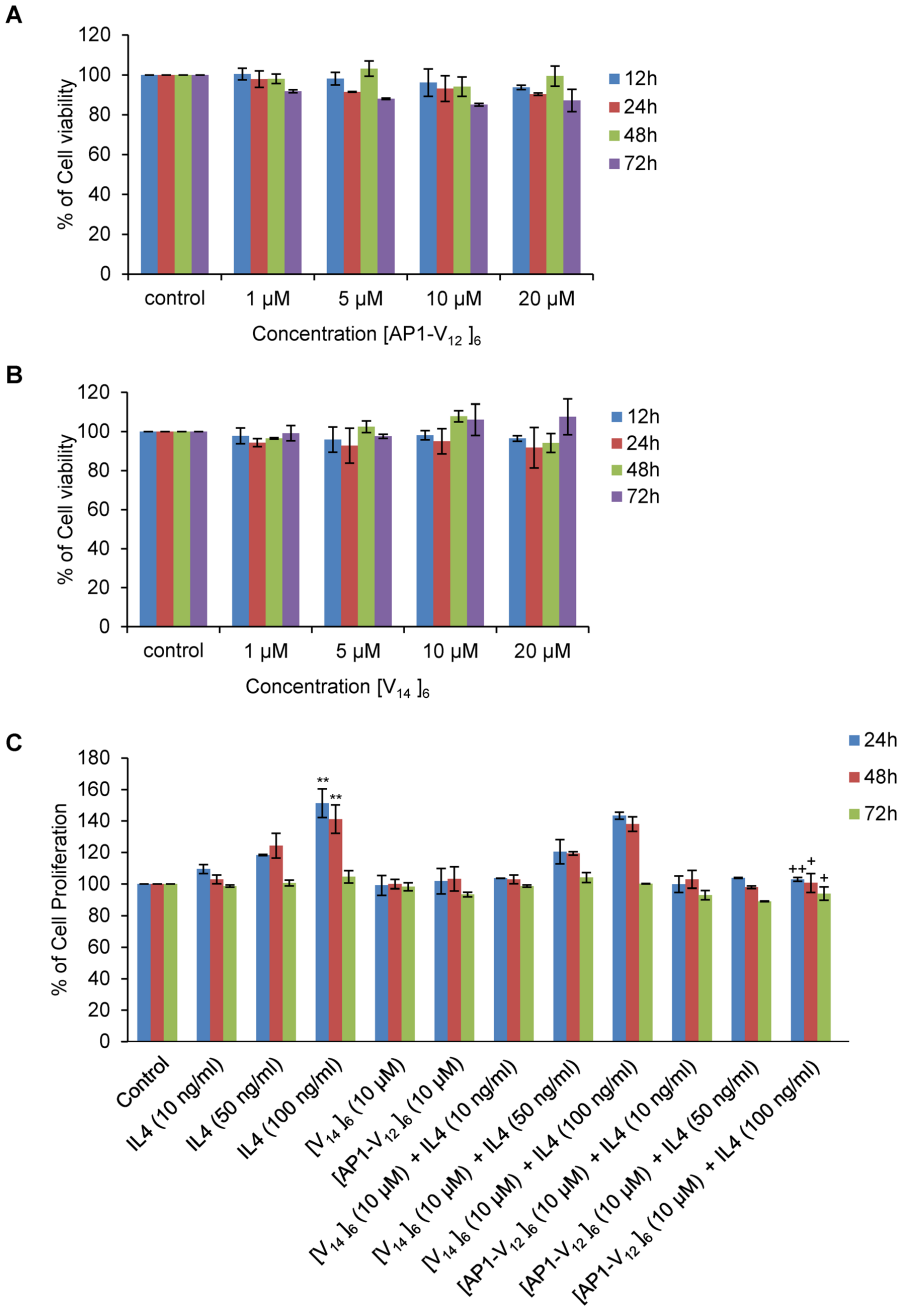
*In vitro* cell viability and proliferation assay. (A, B) MDA-MB-231 cells were plated in 96 well plates in serum (10% FBS) containing media and further treated with different concentration of [AP1-V_12_]_6_ and [V_14_]_6_ (1, 5, 10, 20 µM) for various time intervals (12, 24, 48, and 72 h). Cell viability was accessed by measuring WST-8 absorbance at 450 nm (n = 5) samples at different time intervals. The graph represents the percentage of cell viability in treated cells compared to control (untreated) cells. The result shown here is the representative data of 3 independent experiments. (C) For proliferation assay, MDA-MB-231 (2×10^3^) cells were serum starved (1% FBS) for 16 h with or without different concentrations of IL-4. To check the effect of [AP1-V_12_]_6_ and [V_14_]_6_ on IL-4 stimulation, the cells were grown in low serum (1% FBS) media containing different concentration of IL-4 (10, 50, 100 ng/ml) and [AP1-V_12_]_6_ and [V_14_]_6_ (10 µM) for 24, 48, and 72 h. Cell proliferation was analyzed by measuring the WST-8 absorbance at 450 nm (n = 5 samples). The graph represents the percentage of cell viability in treated cells compared to control (untreated) cells. The result shown here is the representative data of 3 independent experiments. A t-test was performed to determine the significance of various groups after IL-4 and polymer treatments. Control versus IL-4 (100 ng/ml), *  = P<0.05 and **  = P<0.001, IL-4 (100 ng/ml) versus [AP1-V_12_]_6_ + IL-4 (100 ng/ml), +  = P<0.05 and ++  = P<0.001.

The cytotoxic effect of [AP1-V_12_]_6_ and [V_14_]_6_ polymer on proliferation of MDA-MB-231 cells was examined. Cytotoxicity of MDA-MB-231 cells after exposure to increasing concentration of [AP1-V_12_]_6_ and [V_14_]_6_ polymers shown similar pattern of cell viability and proliferation at different time intervals (Fig. 5A, B). Thus, the result indicated that both the polymers are nontoxic to the cancer cell.

We further investigated the effect of [AP1-V_12_]_6_ and [V_14_]_6_ polymer on IL-4 induced cell proliferation in MDA-MB-231 cells. IL-4 (10, 50 and 100 ng/ml) treated cells shown an increased in cell proliferation under low serum condition. The increase in cell proliferation upon IL-4 stimulation was brought to basal level after [AP1-V_12_]_6_ treatment, however cells continue to proliferate in [V_14_]_6_ and IL-4 treated cells (Fig. 5C). Thus, it is very clear that though [AP1-V_12_]_6_ has minimal effect on cell growth, it can inhibit IL-4 mediated cancer cells proliferation probably by binding the IL-4 receptor expressed on cancer cells.

### Surface plasmon resonance analysis to check [AP1-V_12_]_6_ protein affinity for IL-4R

**Figure 6:**
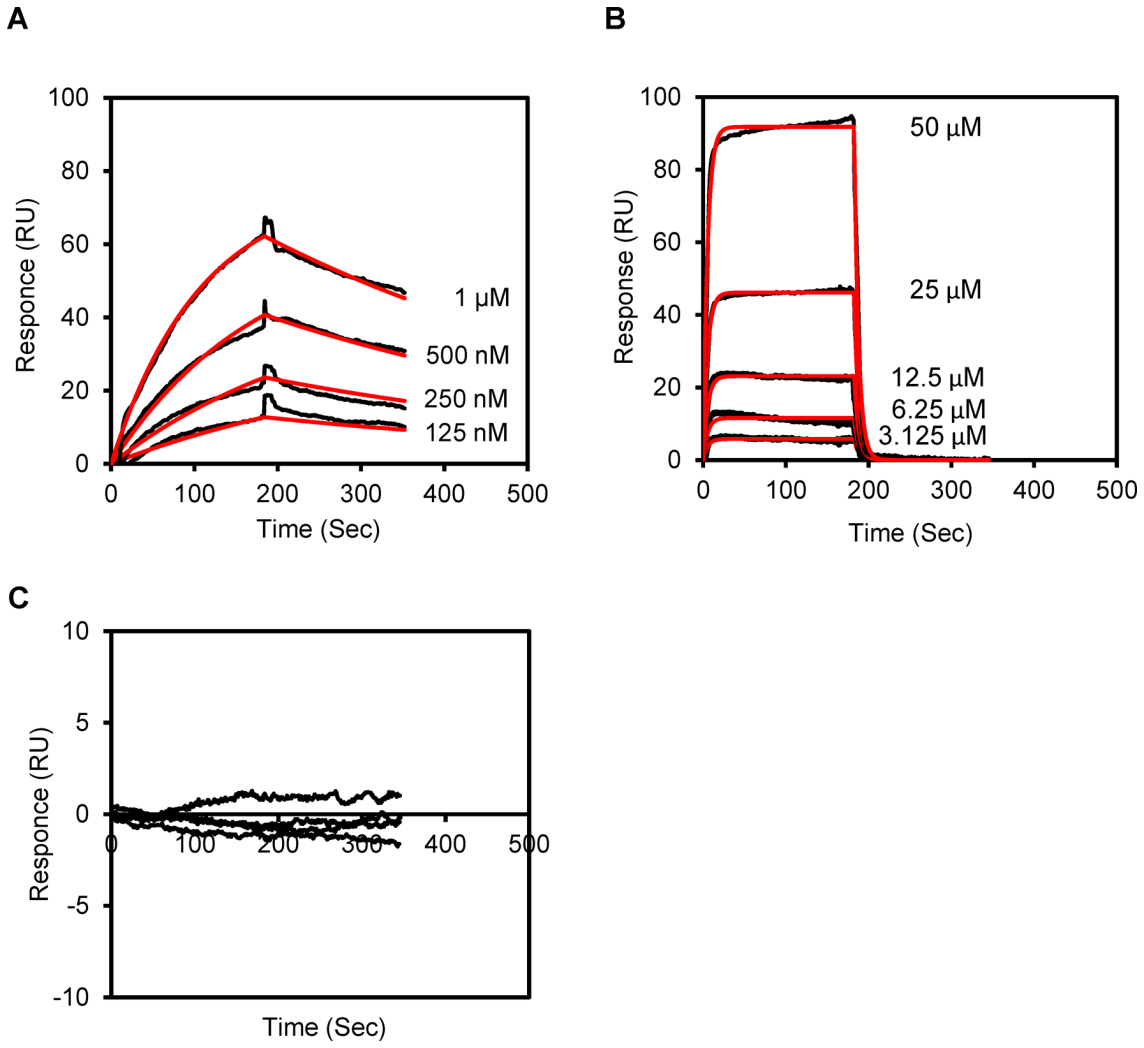
IL-4 R binding affinity of [AP1-V_12_]_6_ polymer. Determination of binding kinetics. Normalized (A) [AP1-V_12_]_6_ protein (125 nM - 1 µM), (B) AP1 peptide (3.125 - 50 µM) and (C) [V_14_]_6_ (1 µM) binding curves (black line) with fits (red line) obtained using Scrubber 2. Histograms are representative of three independent experiments.

The kinetics of AP1 peptide and [AP1-V_12_]_6_ protein binding to the IL-4R were analyzed using surface plasmon resonance [31], [32]. The [AP1-V_12_]_6_ polymer (Fig. 6A) showed higher binding affinity than the AP1 peptide (Fig. 6B) and [V_14_]_6_ (Fig. 6C). The equilibrium constants (K_D_) of free AP1 peptide and [AP1-V_12_]_6_ protein were found to be 5.54±0.3×10^−3^ and 2.07±0.3×10^−7^, respectively (Table 1). Overall, the affinity of the [AP1-V_12_]_6_ polymer for the IL-4R was ∼10000-fold higher than that of free AP1 peptide. No binding of [V_14_]_6_ was detected, even at concentrations as high as 1 µM. Thus, multivalent presentation of the targeting ligand AP1 on the ELP polymer backbone indeed increased the binding affinity towards the IL-4R.

**Table 1:** Determination of binding kinetics.

	k_on_ (M^−1^S^−1^)	k_off_ (S^−1^)	K_D_(M^−1^)
**[AP1-V_12_]_6_**	5.81±1.5×10^3^	1.25±0.5×10^−3^	2.07±0.3×10^−7^
**AP1**	3.5±0.5×10^1^	1.70±0.2×10^−1^	5.54±0.3×10^−3^

_on_), dissociation (k_off_) and equilibrium (K_D_) constants of [AP1-V_12_]_6_ and AP1 peptide from kinetic fits obtained from Scrubber 2. The data ±SD obtained for three independent experiments (n = 3). Association (k

### *In vivo* imaging of [AP1-V_12_]_6_ polymer tumor targeting

**Figure 7:**
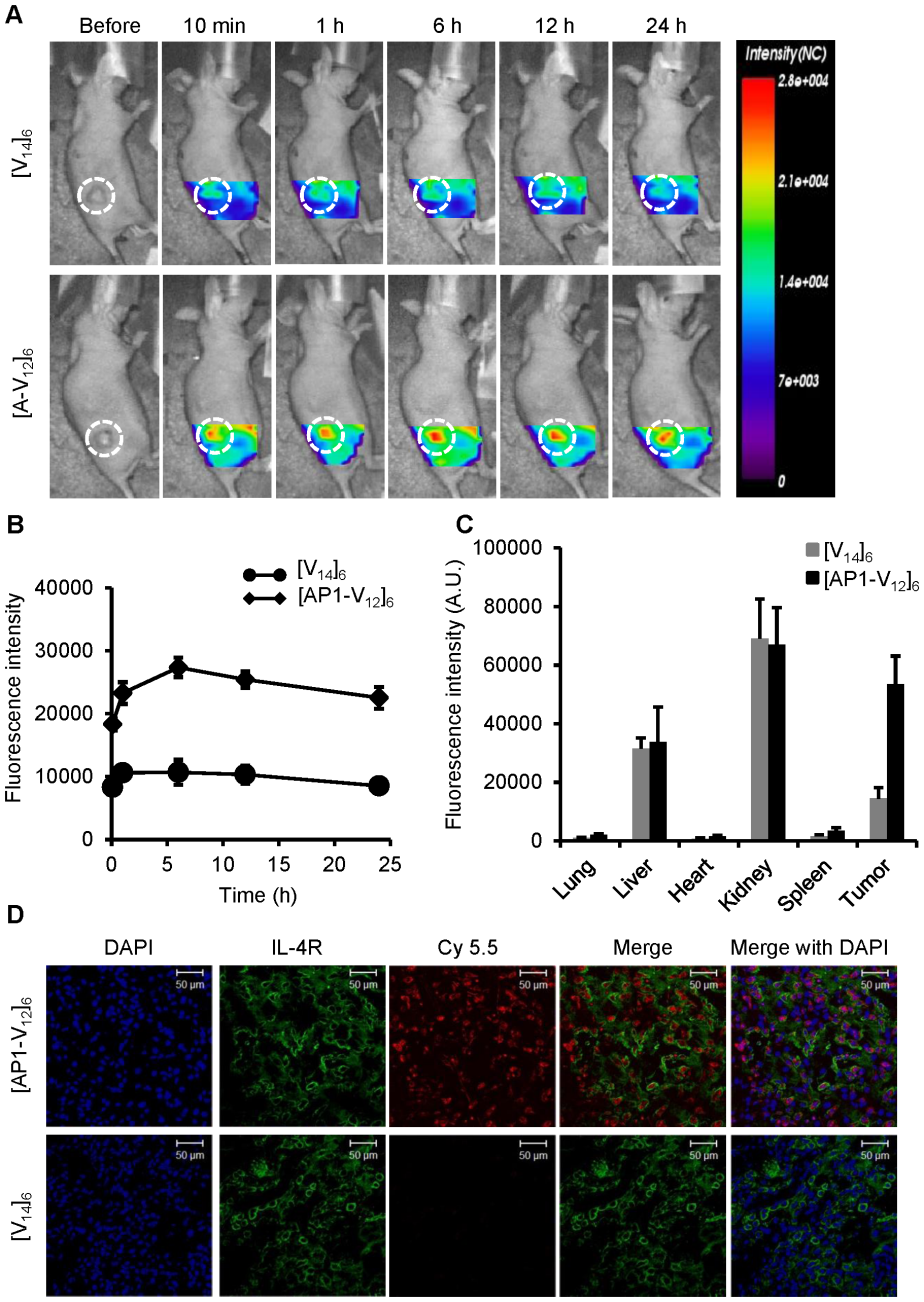
*In vivo*, *ex vivo* imaging and biodistribution of [AP1-V_12_]_6_ polymers. (A) Cy5.5-labeled [V_14_]_6_ and [AP1-V_12_]_6_ (8 mg/kg) were intravenously injected into MDA-MB-231 tumor xenografted nude mice. Biodistribution was determined by collecting *in vivo* fluorescence images at different time points. Scale bar indicates normalized fluorescence intensity (NC). Representative optical images of three experiments. (B) Quantitation of fluorescence intensities in tumor sites at respective time points (n = 3). (C) Analysis of fluorescence intensities for tumors and organs from *ex vivo* images (n = 3). (D) Histological analysis of [AP1-V_12_]_6_ polymer (red) localization in tumors. Nuclei were stained with DAPI (blue), and IL-4R expression on cells was visualized by anti-IL-4 receptor antibody staining (green). Representative confocal images of three experiments (scale bar 50 µm).

The *in vivo* tumor-targeting efficiency of the [AP1-V_12_]_6_ polymer was studied using a near-infrared fluorescence (NIRF) live optical imaging system. *In vivo* fluorescence images taken at different time intervals showed that the [AP1-V_12_]_6_ polymer rapidly accumulated at tumor tissue (within a little as 10 min of injection) in MDA-MB-231 (Fig. 7A, B) breast cancer-bearing mice and was retained in tumor tissues for 24 h after injection. This specific targeting and sustained localization within tumors could provide sufficient time for [AP1-V_12_]_6_ polymer-conjugated drugs to exert their effects. In contrast, [V_14_]_6_ treated mice showed minimal fluorescence intensity at the tumors tissue and more nonspecific tissue localization. Six hours after injection, mice were sacrificed and *ex vivo* fluorescence images of tumor and excised organs were collected to evaluate the biodistribution of [AP1-V_12_]_6_ and [V_14_]_6_. [AP1-V_12_]_6_ fluorescence intensity was higher in the target tumor compared with that of [V_14_]_6_, confirming the increased tumor-targeting specificity and retention time of the AP1 peptide-modified ELP polymer. However, fluorescence intensity was also found to be strong in the liver and kidney of both targeted [AP1-V_12_]_6_ and non-targeted [V_14_]_6_ injected mice, indicating rapid metabolism and secretion in urine (Fig. 7C). An immunohistological examination of tumor tissue showed that abundant staining for [AP1-V_12_]_6_ was confined to the tumor tissue, consistent with our *in vivo* and *ex vivo* imaging results (Fig. 7D).

## Discussion

Cancer cells express a number of cell surface and matrix proteins that mediate tumor growth, migration, invasion, and metastasis. Screening for ligands that specifically target these receptors represents an excellent cancer therapy strategy [33], [34]. Initial studies using phage display technology discovered an IL-4R binding peptide (AP1) that was highly expressed on atherosclerotic plaques and cancer cells [16], [17]. The AP1 peptide was found to bind the IL-4R with low affinity (i.e., micromolar dissociation constant), which means that more targeting peptide is necessary to achieve high receptor occupancy. To improve the binding affinity of AP1 as well as its avidity towards the IL-4 receptor, we incorporated multiple AP1 peptides into the ELP polymer.

The genetically encoded synthesis of [AP1-V_12_]_6_ allows easy synthesis of ligand-presenting ELP polymers and makes it possible to control their molecular weight and T_t_ to conform the requirements of physiological conditions. The ELP polymer backbone is capable of accommodating virtually any target specific ligand and can support multivalent presentation of target ligands without a change in its physical properties [35], [36]. Many researchers have investigated the potential of ELP based incorporation of peptide as targeting moieties and cell penetrating peptide to improve the uptake by cancer cells [37], [38]. Moreover there are many reports on application of diblock ELPs polymers consisting of a hydrophilic block and hydrophobic block capable of forming monodisperse spherical micelles above critical micelle temperature (CMT) for presentation of targeting ligands at their N-terminus site which enables multivalent presentation on their selective targets [36], [39].

In this study for the first time we attempted to design ELP based multivalent targeting polymer by accommodating tandem repeat of highly specific IL-4R binding ligands, AP1 along the ELP polymer to improve its presentation towards IL-4R highly expressed cancer cells. Incorporation of AP1 on ELP elevated the T_t_ compared to ELP alone, likely due to the charged and polar surface residues present on AP1 [30]. Trabbic-Carlson et al. 2004 [30] already reported that the T_t_ of ELP fusion protein was negatively correlated with the fraction of hydrophobic area presented on the surface of the fused folded protein. Proteins with relatively high hydrophobic solvent-accessible surface area depressed the inverse transition temperature of the fused ELP whereas proteins with more hydrophilic surfaces slightly increase elevated the transition temperature of the ELP fusion protein relative to that of the ELP.

The multivalent presentation of the targeting ligand AP1 on the ELP polymer backbone increased binding affinity ∼10000-fold compared to the free AP1 peptide clearly denoted its augmentation in affinity and IL-4R interaction which is important for tumor targeting approach. *In vitro* studies further showed significantly higher binding of the [AP1-V_12_]_6_ polymer to IL-4R highly expressed H226 lung cancer and MDA-MB-231 human breast cancer cells than to IL-4R negative H460 cells. The increased cellular localization of the [AP1-V_12_]_6_ polymer over free AP1 peptide thus verifies the effectiveness of the multivalent, ELP-delivery strategy in improving tumor-targeting activity. Competitive inhibition of [AP1-V_12_]_6_ binding by different concentrations of anti-IL-4R antibody confirmed the specificity towards IL-4R.

Additionally confocal imaging results revealed the increased accumulation and uptake of [AP1-V_12_]_6_ polymer than [V_14_]_6_ control by IL-4R highly expressed tumor cells. Consistent with other findings [4], [40], we believed that the significant enhancement in cellular uptake of thermally responsive [AP1-V_12_]_6_ polymer was due to increased temperature mediated phase transition which could possibly increase the exposure of IL-4R binding site to the cells thereby enhanced localization on cell surface and mediate cellular uptake.

Previous studies have suggested that IL-4 can promote proliferation of various cancer cells of different origin under nutrient depletion stress condition [41]. The experiments were performed to examine the modulating effect of our [AP1-V_12_]_6_ polymer on MDA-MB231 cell proliferation in presence or absence of IL-4. Our cytotoxicity assay revealed that both [AP1-V_12_]_6_ and [V_14_]_6_ polymers are nontoxic to cancer cell, as both polymers have least effect on cell viability or proliferation in absence of external IL-4 supplements. However, under nutrient depletion condition only [AP1-V_12_]_6_ but not [V_14_]_6_ shown inhibition of cell growth in presence of IL-4. Thus our result implicated that [AP1-V_12_]_6_ might inhibit IL-4 mediated cancer cells proliferation probably by binding the IL-4 receptor expressed on cancer cells.

Determination of targeting efficacy *in vivo* is very important for verifying precise homing of [AP1-V_12_]_6_ to tumor tissue. In this study we have used MDA-MB-231 tumor xenograft mouse model because of rapid propagation of cells subcutaneously, re-forming into solid tumors within a short duration. *In vivo* fluorescence imaging of the tumor site at different times after injection confirmed higher levels of fluorescence signals of [AP1-V_12_]_6_ in MDA-MB-231 tumor-bearing mice, an observation that was further confirmed by immunohistological examinations of frozen tissue sections. Previous *in vivo* studies using pH-responsive, AP1-containing micelles demonstrated gradual binding to tumor tissue with maximum intensity at 24 h [18]. Notably, our genetically engineered ELP-based AP1 polymers further enhanced tumor targeting ability, showing high tumor accumulation that was evident as early as 10 min after intravenous injection, peaked after about 6 h, and was maintained up to 24 h (Fig. 7). With the exception of kidney and liver, fluorescence levels were negligible in off-target organs, including the lung, spleen, and heart. Even though our designed the [AP1-V_12_]_6_ polymer has T_t_ just above the physiological body temperature, which is preferable for clinical applications [42], T_t_ of [V_14_]_6_ control is found to be relatively lower than the [AP1-V_12_]_6_. However, the difference in T_t_ was not an emphasis due to application of lesser concentration of non-targeting and targeting polymer during *in vivo* experiments. Besides, [V_14_]_6_ control was found to accumulate in various organs post intravenous injection except in the tumor tissue unlike the case of [AP1-V_12_]_6_ injected groups with precise localization to tumor tissue. Collectively, these findings highlight the promise of our [AP1-V_12_]_6_ polymer as a candidate macromolecular drug delivery system. Although our ELP-based polymers can target only IL-4R overexpressed cancer cells, many tumor of different origin have been reported to highly expressed IL-4R [19]–[22] and thus our designed polymer can be used as a potent drug carrier for various targeted cancer therapies.

The strategy of designing macromolecular carrier systems with multivalent ligand presentation for targeting surface molecules displayed on solid tumors has clear value in clinical applications. Because of relatively simple and novel synthetic process and the ability to use multivalent peptides and targeting moieties, we proposed a method which is distinctive and explore a new arena of multivalent cancer targeting. Although our multivalent targeting system exhibited effective tumor accumulation, further study is needed to realize the promise of ELP-based targeting polymers as carriers for delivering chemotherapeutic agents and therapeutic peptides.

## Supporting Information

Figure S1 **Agarose gel electrophoresis of (A) [V_14_]_n_ and (B) [AP1-V_12_]_n_, (n = 1, 2, 4, 5 and 6) represents the number of monomer-gene repeats, visualized by ethidium bromide staining.** The left lane contains a 1-kb DNA size marker. pRSET vectors containing ELP genes were double digested with *Bam*H I and *Hin*D III, producing two bands corresponding to the vector (2900 bp) and variable-sized ELPs. The number of monomer-gene repeats (1, 2, 4, 5 and 6) is labeled on top, and expected ELP sizes are given on the right. (C) Partial sequence consists of *Sfi* I site, used for pET25b+ vector modification. (D) [V_14_]_6_ and (E) [AP1-V_12_]_6_ genes digested with *Pfl*M I and *Bgl* I were ligated to *Sfi* I-digested, modified pET 25b+ vector. The sizes of DNAs were confirmed by double digestion with *Nde* I and *Hin*D III. Two bands corresponding to the vector (5547 bp) and ELP gene (1294 bp) were produced.
(TIFF)

Figure S2 **Confocal microscopic images of MDA-MB-231 cells treated with 10 µM of [AP1-V_12_]_6_, AP1, or [V_14_]_6_ for 1 h at (A) 4°C and (B) 37°C.** Unbound peptides were washed out with PBS, and cells were fixed with 4% paraformaldehyde. Cell nuclei were stained with DAPI. Representative confocal images of three experiments (scale bar 20 µm).
(TIFF)

Figure S3 **Confocal microscopic images H460 cells treated with 10 µM of [AP1-V_12_]_6_, AP1, or [V_14_]_6_ for 1 h at (A) 4°C and (B) 37°C.** Cells were fixed with 4% paraformaldehyde and cell nuclei were stained with DAPI. Representative confocal images of three experiments (scale bar 20 µm).
(TIFF)
