# Supplementary figures and images for: Construction and Application of Elastin Like Polypeptide Containing IL-4 Receptor Targeting Peptide
Source: PLoS One. 2013 Dec 10;8(12):e81891. doi: 10.1371/journal.pone.0081891 (PMC3858272; doi:10.1371/journal.pone.0081891)

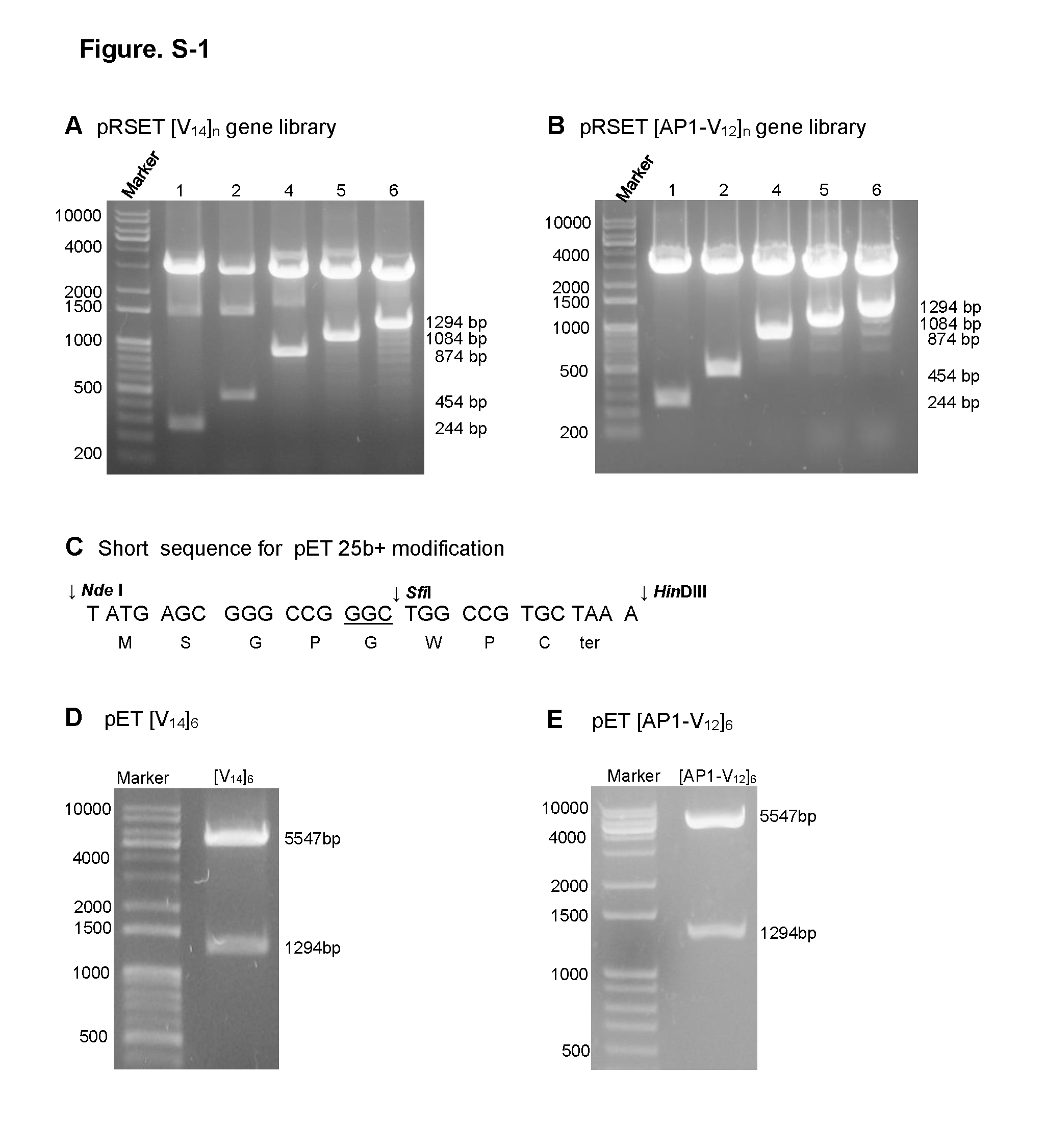

Supplement: Figure S1 — Agarose gel electrophoresis of (A) [V14]n and (B) [AP1-V12]n, (n = 1, 2, 4, 5 and 6) represents the number of monomer-gene repeats, visualized by ethidium bromide staining. The left lane contains a 1-kb DNA size marker. pRSET vectors containing ELP genes were double digested with BamH I and HinD III, producing two bands corresponding to the vector (2900 bp) and variable-sized ELPs. The number of monomer-gene repeats (1, 2, 4, 5 and 6) is labeled on top, and expected ELP sizes are given on the right. (C) Partial sequence consists of Sfi I site, used for pET25b+ vector modification. (D) [V14]6 and (E) [AP1-V12]6 genes digested with PflM I and Bgl I were ligated to Sfi I-digested, modified pET 25b+ vector. The sizes of DNAs were confirmed by double digestion with Nde I and HinD III. Two bands corresponding to the vector (5547 bp) and ELP gene (1294 bp) were produced. (TIFF) [file pone.0081891.s001.tiff]

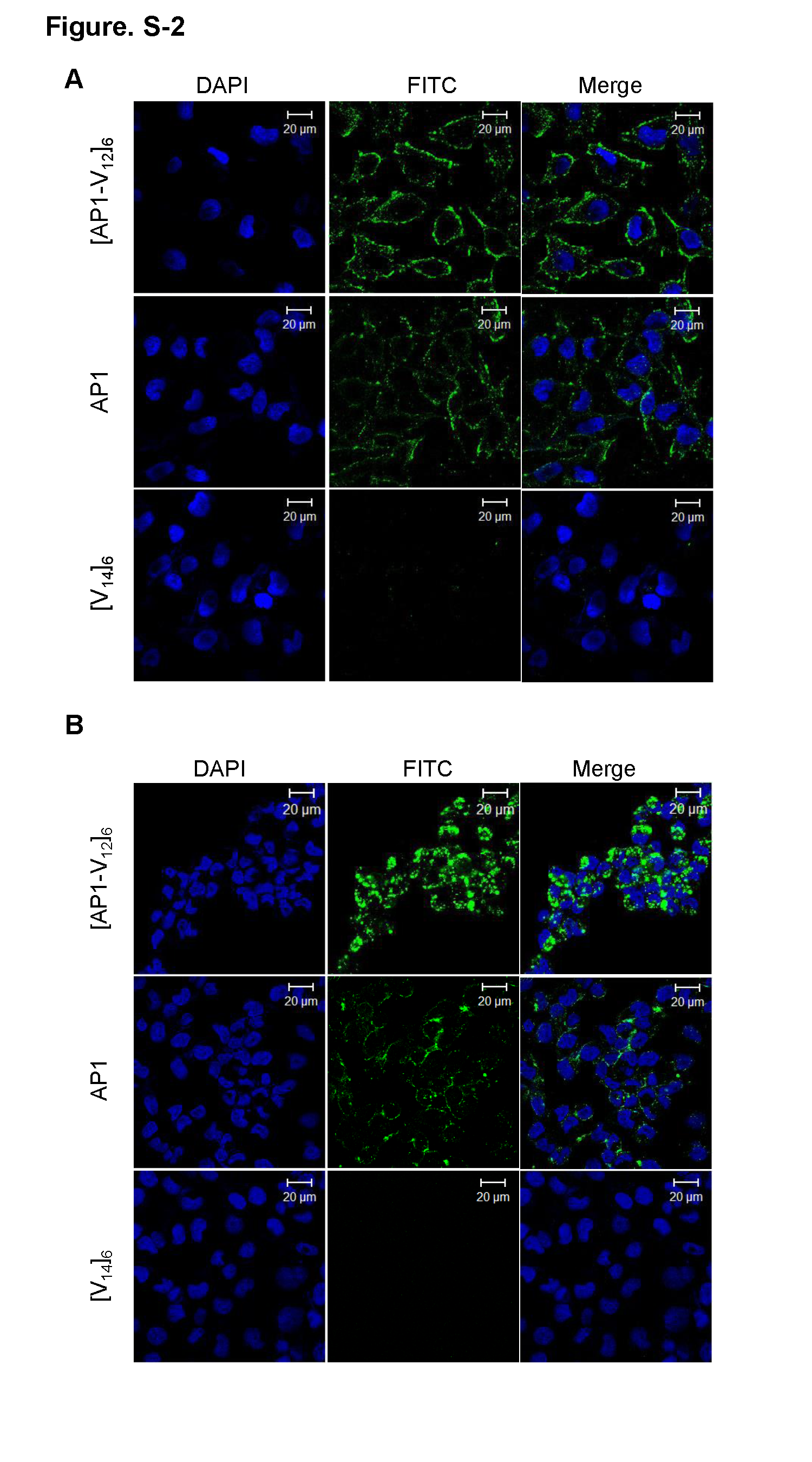

Supplement: Figure S2 — Confocal microscopic images of MDA-MB-231 cells treated with 10 µM of [AP1-V12]6, AP1, or [V14]6 for 1 h at (A) 4°C and (B) 37°C. Unbound peptides were washed out with PBS, and cells were fixed with 4% paraformaldehyde. Cell nuclei were stained with DAPI. Representative confocal images of three experiments (scale bar 20 µm). (TIFF) [file pone.0081891.s002.tiff]

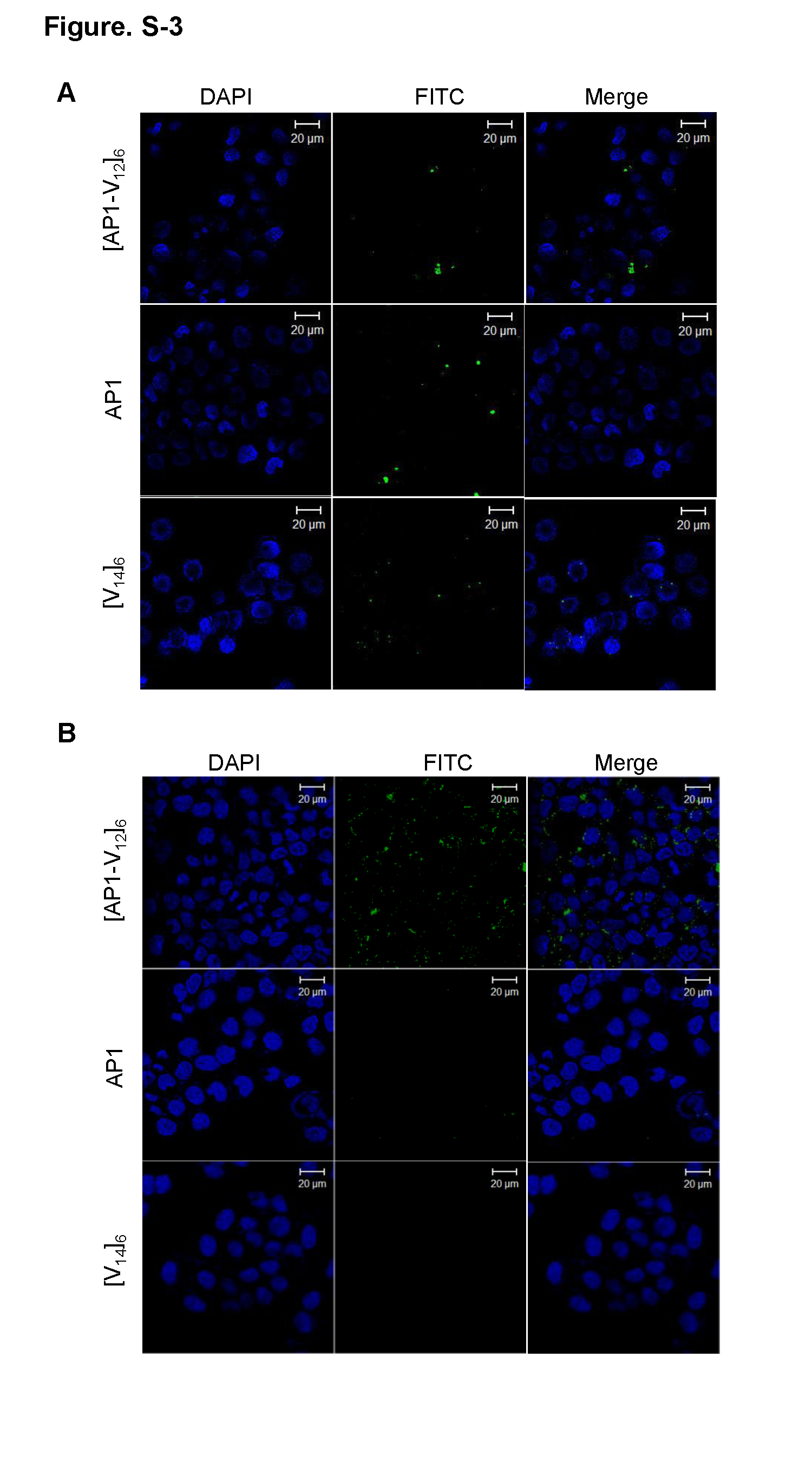

Supplement: Figure S3 — Confocal microscopic images H460 cells treated with 10 µM of [AP1-V12]6, AP1, or [V14]6 for 1 h at (A) 4°C and (B) 37°C. Cells were fixed with 4% paraformaldehyde and cell nuclei were stained with DAPI. Representative confocal images of three experiments (scale bar 20 µm). (TIFF) [file pone.0081891.s003.tiff]
